# Microbial community dynamics and effect of environmental microbial reservoirs on red-backed salamanders (*Plethodon cinereus*)

**DOI:** 10.1038/ismej.2013.200

**Published:** 2013-12-12

**Authors:** Andrew H Loudon, Douglas C Woodhams, Laura Wegener Parfrey, Holly Archer, Rob Knight, Valerie McKenzie, Reid N Harris

**Affiliations:** 1 Department of Biology, James Madison University, Harrisonburg, VA, USA; 2 Department of Ecology and Evolutionary Biology, University of Colorado, Boulder, CO, USA; 3 BioFrontiers Institute, Universitys of Colorado, Boulder, CO, USA; 4 Howard Hughes Medical Institute, University of Colorado, Boulder, CO, USA

**Keywords:** amphibians, bacterial reservoirs, *Batrachochytrium dendrobatidis*, community dynamics, host–bacteria interactions, symbiosis

## Abstract

Beneficial cutaneous bacteria on amphibians can protect against the lethal disease chytridiomycosis, which has devastated many amphibian species and is caused by the fungus *Batrachochytrium dendrobatidis*. We describe the diversity of bacteria on red-backed salamanders (*Plethodon cinereus*) in the wild and the stability of these communities through time in captivity using culture-independent Illumina 16S rRNA gene sequencing. After field sampling, salamanders were housed with soil from the field or sterile media. The captive conditions led to different trajectories of bacterial communities. Eight OTUs present on >90% of salamanders in the field, through time, and in both treatments were defined as the core community, suggesting that some bacteria are closely associated with the host and are independent of an environmental reservoir. One of these taxa, a *Pseudomonas sp.*, was previously cultured from amphibians and found to be antifungal. As all host-associated bacteria were found in the soil reservoir, environmental microbes strongly influence host–microbial diversity and likely regulate the core community. Using PICRUSt, an exploratory bioinformatics tool to predict gene functions, we found that core skin bacteria provided similar gene functions to the entire community. We suggest that future experiments focus on testing whether core bacteria on salamander skin contribute to the observed resistance to chytridiomycosis in this species even under hygenic captive conditions. For disease-susceptible hosts, providing an environmental reservoir with defensive bacteria in captive-rearing programs may improve outcomes by increasing bacterial diversity on threatened amphibians or increasing the likelihood that defensive bacteria are available for colonization.

## Introduction

Host-associated bacterial communities affect health in many species, including humans (Fierer *et al.*, 2012), corals (Rosenberg *et al.*, 2007), insects (Dillon *et al.*, 2005) and amphibians (Harris *et al.*, 2009a, 2009b). The cutaneous microbial community of amphibians provides a defensive function against pathogens, including the fungus *Batrachochytrium dendrobatidis* (*Bd*) (Woodhams *et al.*, 2007; Becker and Harris, 2010). *Bd* causes the fungal disease chytridiomycosis and has caused global amphibian extinctions and population declines (Berger *et al.*, 1998; Lips *et al.*, 2006; Rachowicz *et al.*, 2006; Skerratt *et al.*, 2007; Crawford *et al.*, 2010). Previous studies have not examined the source of the bacteria or the temporal dynamics of amphibians' defensive bacterial communities. In order to understand the association between microbiota and health, we must first characterize the microbial community and its variation through time. We experimentally examined the stability and diversity of red-backed salamander (*Plethodon cinereus*) microbiota through time under different environmental conditions.

As amphibians' microbiota produces antifungal metabolites, the stability of the microbiota may be critical to amphibian health. A fluctuating community structure may result in a fluctuating defensive function, whereas a stable microbiota may provide more continual protection from pathogens. An amphibian's protective microbial community may not be stable because of perturbations such as skin sloughing or seasonal temperature changes. Past surveys of amphibians' cutaneous bacteria have only encompassed one time point (Lauer *et al.*, 2007, 2008; Woodhams *et al.*, 2007).

Stability of amphibians' cutaneous microbiota likely depends on bacterial reservoirs such as soil or water for re-colonization (Belden and Harris, 2007). Soil contains a high bacterial diversity (Lauber *et al.*, 2009) and a high abundance of microbes (Whitman *et al.*, 1998). For example, 1 g of soil is estimated to contain between 10^6^ and 10^9^ bacterial cells (Whitman *et al.*, 1998) and is among the richest environmental substrates for bacterial diversity (Lauber *et al.*, 2009; Fierer and Lennon, 2011). Furthermore, terrestrial salamanders, such as red-backed salamanders, are in constant contact with soil.

Understanding the importance of environmental reservoirs for the hosts' microbial community structure has consequences for ecological studies conducted in the laboratory (for example, Becker *et al.*, 2009; Becker and Harris, 2010; Woodhams *et al.*, 2012) and for conservation because endangered amphibians are often brought into survival assurance colonies, which removes the animals from their natural environment. Typically, the laboratory environment lacks natural bacterial reservoirs, which might strongly affect microbial structure, diversity and function of the skin microbiota. Thus, removal from a natural environment is likely to be a major perturbation for the amphibian microbiota and may have impact on the capacity for the microbiota to defend against disease. It is unknown how this may affect disease susceptibility in the laboratory environment or in the wild following release.

We tested the hypothesis that a natural soil bacterial reservoir was required to maintain the stability and diversity of bacterial communities on salamanders through time by sampling salamanders in the field and then after captive-housing with or without a bacterial reservoir. We examined alpha diversity, which measures the bacterial community diversity on individual salamanders, and beta diversity, which measures the difference among bacterial communities. In addition, we tested the hypothesis that salamanders exposed to a perturbation retained a core community. Finally, we tested whether predicted functions of the core community differed significantly from those of the non-core community.

## Materials and methods

### Experimental design

Red-backed salamanders (*P. cinereus*) were chosen for this study because their bacterial communities have been extensively studied with respect to their capacity to protect them against chytridiomycosis (Becker *et al.*, 2009; Harris *et al.*, 2009b; Becker and Harris, 2010). In addition, *P. cinereus* is abundant in the Shenandoah mountain region of Virginia, are in close contact with soil and tolerate laboratory conditions. Salamanders were collected from the George Washington National Forest in October 2011 (VADGIF Permit No. 047519), and the soil in this experiment was collected at the same location and time. The JMU IACUC approved our experimental protocol.

After collection, each salamander was immediately rinsed with sterile Provasoli media three times to remove transient bacteria (Lauer *et al.*, 2007; McKenzie *et al.*, 2011). New gloves were used between each salamander. The salamanders were swabbed 10 times on a randomly chosen left or right side of their ventral surface with a sterile rayon swab (BBL CultureSwab, BD Diagnostics, Franklin Lakes, NJ, USA). The bacterial community of the immediate environment for each salamander was also sampled by being swabbed with 10 strokes back and forth. All samples were stored on ice and then frozen at −80 °C until DNA extraction.

Salamanders were transported to the laboratory in sterile 50-ml falcon tubes (BD Diagnostics), and the soil was transported in autoclaved plastic containers. Salamanders were housed individually in 17 cm × 12 cm × 7 cm (L × W × H) plastic containers kept at 17 °C on a 12-h-light- and 12-h-dark cycle. Salamanders were randomly assigned to one of two treatments to test the hypothesis that the type of environmental reservoir affects community stability and composition. The ‘sterile media' treatment (*n*=10) consisted of 30 ml of sterile Provasoli medium (Wyngaard and Chinnappa, 1982). The ‘soil' treatment (*n*=10) consisted of 150 g of soil from the salamanders' natural habitat. The soil was homogenized by hand with sterile gloves prior to its placement in the salamander containers, and initial soil samples were taken in triplicate for bacterial community identification. Salamanders were swabbed every 7 days until day 28 (Table 1). Media were replaced every 7 days following sampling; soil was not replaced. Soil was sampled in triplicate on days 0, 14 and 28 from salamanders in the soil treatment. Each salamander was fed 15 fruitflies once a week after sampling took place. The bacteria associated with fruitflies likely did not contribute meaningfully to the microbes in the system, given the bacterial biomass in soil; however, they may have been a source of bacteria not present in the soil.

### Molecular techniques

DNA extractions and 16S rRNA amplification were performed according to Caporaso *et al.,* (2012) and the EMP protocol (http://www.earthmicrobiome.org/emp-standard-protocols/). Samples, along with aliquots of the sequencing primers, were processed on an Illumina HiSeq 2000 (Caproaso *et al.*, 2011, 2012) at the Biofrontiers Next-Gen Sequencing Facility located at the University of Colorado, Boulder, USA. An aliquot of the DNA was also used to test each salamander for the presence of *Bd* using the standard PCR protocol (Annis *et al.*, 2004).

These data, along with MiMARKs compliant metadata (http://gensc.org/gc_wiki/index.php/MIMARKS), are available in the Quantitative Insights Into Microbial Ecology (QIIME) database (www.microbio.me/qiime; study no. 1618). Data have been deposited at the European Bioinformatics Institute (EBI) archive with the accession number ERP003771.

### Sequence analysis

Amplicons were sequenced on 1/3 of an Illumina HiSeq lane at the University of Colorado at Boulder yielding 100-bp reads. Quantitative Insights Into Microbial Ecology (QIIME) version 1.5.0 (Caporaso *et al.*, 2010b) was used for all sequence analysis, unless otherwise noted. Sequences were filtered for quality and assigned to their respective sample using default settings. The resulting 23.3 million reads were clustered into operational taxonomic units (OTUs) according to the subsampling open reference protocol using the October 2012 release of Greengenes (McDonald *et al.*, 2012) with reference sequences clustered at 97% (available at www.greengenes.secondgenome.com). In total, 83% of the reads hit the reference data set and were assigned to reference OTUs, which used the reference Greengenes taxonomy. The remaining sequences were clustered into *de novo* OTUs and taxonomy was assigned to these using the RDP classifier retrained on the Greengenes 2012 data with 80% confidence threshold. OTUs with fewer than 100 reads were filtered out of our analysis (Bokulich *et al.*, 2013), resulting in a total of 21.7 million sequences clustered into 6049 OTUs. Sequences were aligned to the Greengenes reference alignment using PyNAST (Caporaso *et al.*, 2010a), and a phylogenetic tree was constructed with FastTree according to the standard procedures within QIIME (Price *et al.*, 2009). Samples with fewer than 6000 sequences were removed from the analysis; 40 out of 183 samples were removed at this step. Alpha and beta diversity analyses were conducted on data rarefied to 6300 sequences per sample. To determine whether there were treatment effects on alpha diversity indices (richness and Shannon diversity index), Linear mixed models were performed using the software IBM SPSS Statistics v. 21 (Armonk, NY, USA). Beta diversity was calculated with QIIME using the weighted metrics (Lozupone *et al.*, 2007). The resulting distance matrices were imported into PRIMER 6 (Clarke and Gorley, 2006) for further analysis. The treatment and temporal effects were statistically analyzed using a permutational multivariate analysis of variance, considering time as a random effect and treatment as the fixed effect and were visualized using Principal Coordinates Analysis (PCoA); weighted UniFrac was used for this analysis, as it takes into account the relative abundance in addition to the presence of bacterial taxa. Unweighted UniFrac analyses gave the same results and are therefore not presented.

To test for evidence of a core bacterial community on the skin of red-backed salamanders, we used the compute_core_microbiome function within QIIME, requiring the core OTUs to be present in at least 90% of the samples on the non-rarefied data set. This cutoff was used in previous studies of core human skin bacteria (Caporaso *et al.*, 2011). The mean relative abundance was determined for the resulting eight OTUs and a heat map was created to visualize changes through time and treatments. We also included *Janthinobacterium lividium* in the heat map, as this taxon has previously been shown to produce antifungal metabolites (Brucker *et al.*, 2008), and it was found on a large proportion of the salamanders in the field sample. To determine whether alpha diversity and the relative abundance of the core OTUs differed between treatments, a linear mixed model was used in SPSS. We used Pearson correlations to assess associations between diversity on salamanders and reservoirs. We determined the taxonomy of OTUs of interest by constructing a maximum likelihood tree and comparing them with Greengenes reference sequences.

To explore the functional profiles of our bacterial community data set, we used a bioinformatics tool that predicts gene family abundances based on 16S gene surveys, given a database of phylogenetically referenced genomes (PICRUSt, Phylogenetic Investigation of Communities by Reconstruction of Unobserved States (http://picrust.github.com, 3 July 2013) Langille *et al.*, 2013). This analysis works from the observation that there is an association between phylogeny and gene content. For the analysis, OTUs were closed-reference picked against the 18 May 2012 Greengenes database using QIIME v 1.7 according to the online protocol. The resulting data set was rarefied at 4600 16S rRNA sequences per sample. We predicted the metagenome for each of our samples, as well as the metagenome for the core set of bacteria found in at least 80% of samples, the threshold chosen to include the anti-Bd bacterium *J. lividum* as well as *Pseudomonas viridiflava* and five other phylotypes. PiCRUSt requires that OTUs should be present in the reference database; thus, the novel Verrucomicrobia OTU was excluded from this analysis. We used these data to assess whether the gene functions provided by the common core bacteria differ significantly from the functions provided by the complete community in field-collected skin samples.

The accuracy of metagenome predictions depends on how closely related the microbes in a given sample are to microbes with sequenced genome representatives, as measured by the Nearest Sequenced Taxon Index (NSTI), with lower values indicating a closer mean relationship (Langille *et al.*, 2013). Salamander samples had good NSTI values of 0.09±0.02, and the core samples had values of 0.07±0.02. For comparison, Langille *et al.* (2013) found that human-associated samples had the lowest (best) NSTI values (0.03±0.2). Other mammalian guts had a higher mean NSTI value (0.14±0.06), and diverse communities such as soil also had a much higher NSTI value (0.17±0.02). Thus, the salamander skin samples provide an ideal data set to examine predictions from PICRUSt.

## Results

All environmental samples and 65 out of 100 salamander samples were successfully amplified. Two out of three negative control swabs had no extracted DNA as expected. However, one negative control was apparently contaminated with field soil samples; their community profiles were similar. All salamanders tested were negative for *Bd*.

Having a soil reservoir strongly affected cutaneous microbial diversity on salamander skins. Alpha diversity, as measured by the Shannon diversity index and by OTU richness (similar results not shown), was initially similar across treatments but diverged over time. The Shannon diversity index decreased over time in the sterile media treatment (F_1, 30.587_=128.734; *P*<0.001; Figure 2). Day of the experiment was also a major factor affecting diversity (F_4, 21.103_=25.038; *P*<0.001), and there was a significant interaction between treatment and day (F_4, 21.103_=12.3; *P*<0.001).

The alpha diversity of a salamander's environment at the time of sampling did not predict the diversity of the salamander microbiota in the field (*r*=−0.14792; *P*=0.5256). In addition, there was no correlation between the alpha diversity of the salamanders' microbes on days 0 and 28 for salamanders in the sterile media treatment (*r*=−0.118; *P*=0.802) or for salamanders in the soil treatment (*r*=−0.218; *P*=0.972).

**Figure 1:**
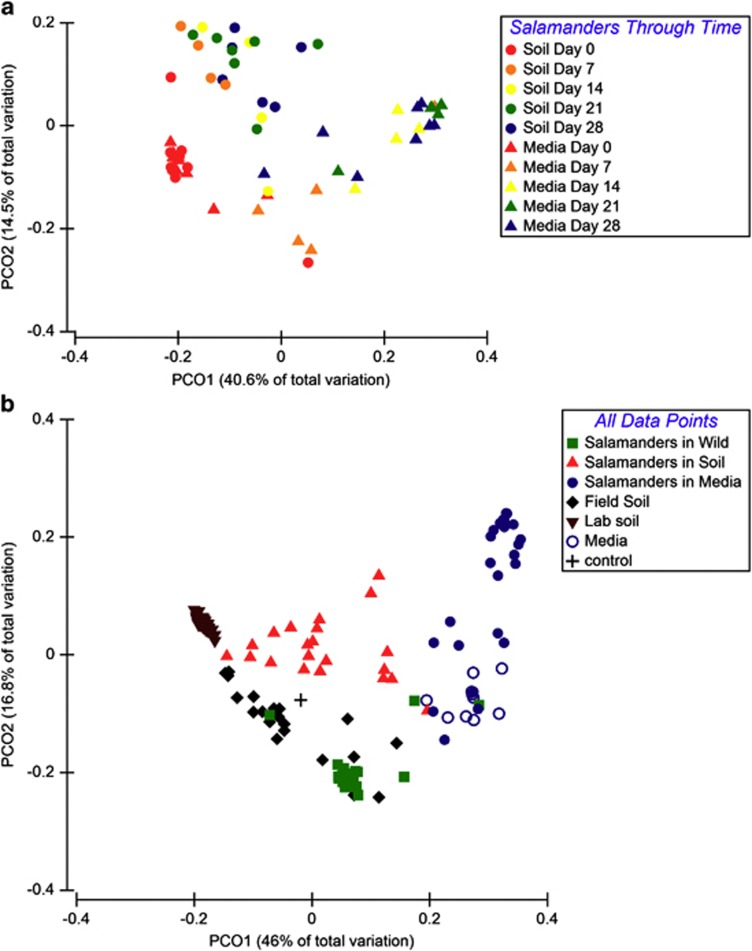
Principal coordinates illustrating similarity between bacterial communities. (**a**) Principal coordinate plot of salamanders in each treatment (media and soil) through time. Each point represents a bacterial community from one red-backed salamander. Salamanders housed with soil (bacterial reservoir) are denoted by circles and salamanders housed with Provasoli media are denoted by triangles. Color indicates the day of sampling. (**b**) Principal coordinates plot of all samples. Each point represents a bacterial community from the environment or on one red-backed salamander. Green triangles represent salamanders housed with a bacterial reservoir. Red squares represent salamanders housed without a bacterial reservoir. Black upside-down triangles represent laboratory soil (the bacterial reservoir) and brown diamonds represent field soil. Blue circles represent media.

Using weighted UniFrac analysis, microbial communities shifted from their original composition in the field when moved into the laboratory, and the treatments led to different microbial community structures (Figure 1a). Bacterial communities in the two treatments were significantly different (Pseudo-F_(1,4.18)_=7.852; *P*=0.031), the effect of time in captivity was significant (Pseudo-F_(4,55)_=7.702; *P*=0.001), and there was an interaction between treatment and the day of experiment (Pseudo-F _(4,55)_=2.867; *P*=0.001). The bacterial communities on salamanders without a bacterial reservoir had fewer OTUs and were often dominated by Verrucomicrobia.

Community structures between microbial communities in field soil, laboratory soil and in media differed (Pseudo-F_(2,87)_=77.7; *P*<0.001). The community structure found in the laboratory soil changed over the course of the experiment (Pseudo-F_(2,59)_=14.092; *P*=0.001). All data points are presented in Figure 1b. Importantly, as the communities on salamanders housed with or without a bacterial reservoir diverged, they became more similar to their respective substrates.

**Figure 2:**
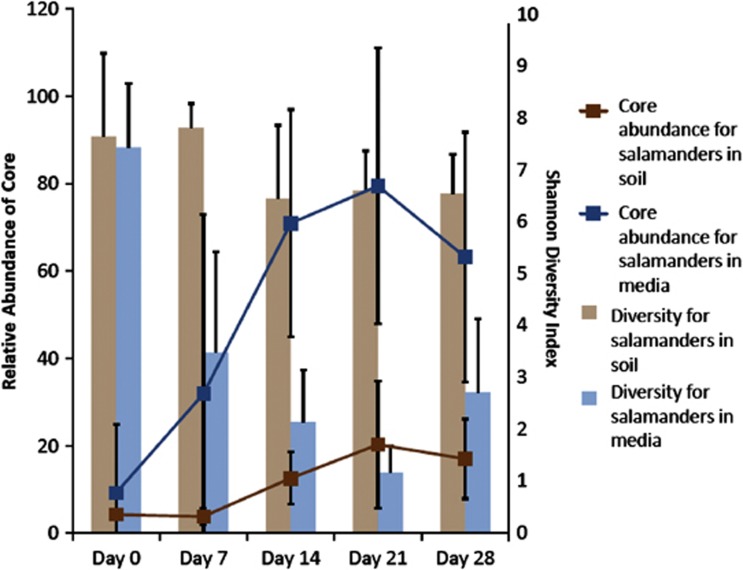
The average proportional relative abundance of all eight core OTUs and the alpha diversity (Shannon diversity index) of all salamanders through the course of the experiment. As alpha diversity decreased, the abundance of the core OTUs increases.

A core community consisted of eight OTUs that were found on >90% of salamanders in the field and through all time points in the experiment in both treatments (Table 2). The relative abundance of the core community increased (F_1,26.182_=40.982; *P*<0.001) and did so more in the sterile media treatment over time than in the soil treatment (interaction between treatment and day F_4, 21.771_=6.856; *P*<0.01) (Figure 2). In the field, the core community comprised of a small fraction of the core community, and it remained so in the soil treatment through time. However, the core community comprised as much as 93.5% of the total community on day 21 of the experiment in the sterile media treatment (Figure 2). The OTU in the phylum Verrucomicrobia often became relatively the most abundant, and it greatly increased in the sterile media treatment, comprising as much as 92.5% of the entire community. Day of the experiment also greatly affected the relative abundance of the core community (F_4,21.771_=15.926, *P*<0.001). There was a negative correlation of alpha diversity and the abundance of the Verrucomicrobia OTU (*r*=−0.843; *P*<0.0001), and a negative correlation between the abundance of the core and the alpha diversity (*r*=−0.883; *P*<0.0001). The antifungal bacterium *J. lividum,* which has been found on *P. cinereus* in previous studies and has been used successfully as a probiotic (Lauer *et al.*, 2007; Harris *et al.*, 2009a), was also a prevalent community member. *J. lividum* was found on 94% of salamanders in the field (day 0) and on 87% of salamanders in both treatments over time, and its relative abundance did not change over time (Fisher's exact test, *P*>0.05). In addition, five out of the eight core OTUs were *Pseudomonadaceae*, some members of which are easily and commonly cultured from red-backed salamanders (Lauer *et al.*, 2007, 2008; Woodhams *et al.*, 2007).

The most prevalent and abundant OTU, found in 100% of samples, was a novel member of the phylum Verrucomicrobia. To gain better taxonomic resolution for this OTU, we constructed a maximum likelihood phylogenetic tree by placing the novel Verrucomicrobia sequences within the Greengenes reference tree (filtered to sequences with 85% similarity; obtained from http://greengenes.secondgenome.com/downloads/database/12_10) using the EPA algorithm within RAxML (Berger and Stamatakis, 2011). This OTU was robustly placed within the Verrucomicrobia class Opitutuae (Supplementary Figure 1).

**Figure 3:**
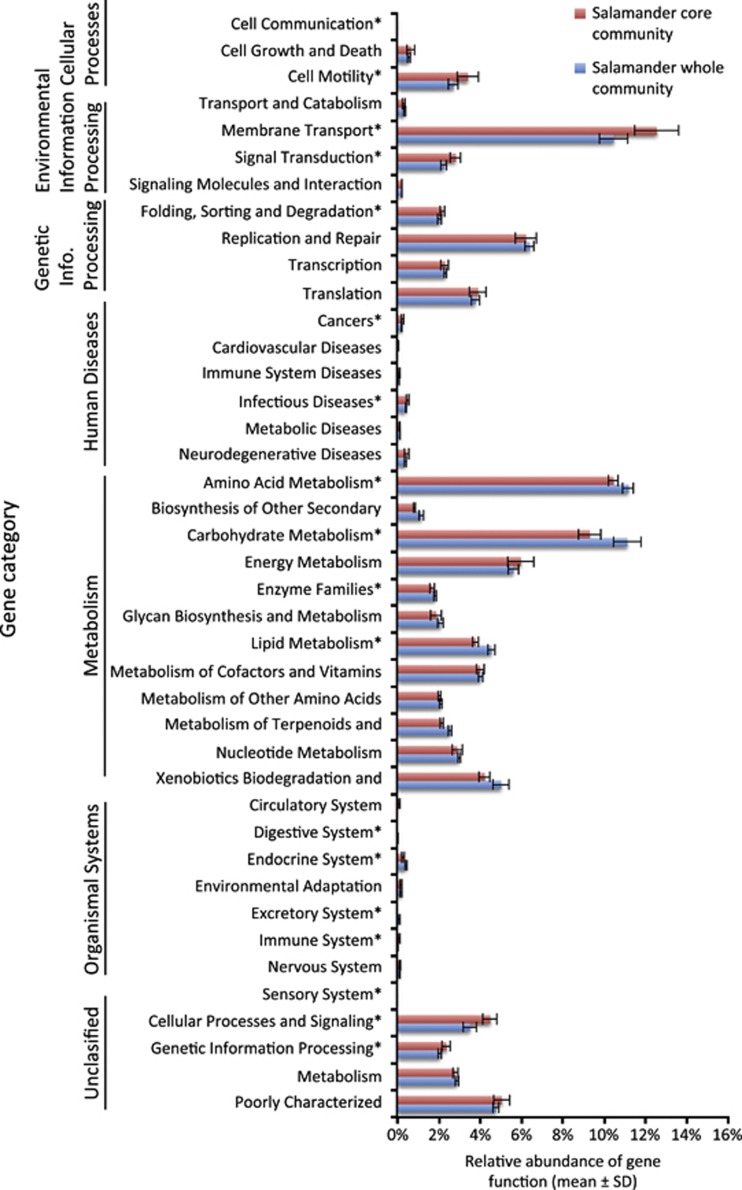
Predicted functions of the bacterial communities found on salamander skin (sampled in field, day 0). * indicates gene categories that are significantly different (*t*-test, Bonferroni-corrected *P*<0.05) between the whole community and the core bacterial OTUs present on >80% of salamanders including *Pseudomonas viridiflava* and *Janthinobacterium lividum* and five other phylotypes.

Using PICRUSt as a predictive exploratory tool, we found that overall 41 of 43 level 2 KEGG Orthology groups (KOs) were represented in the data set. When comparing the function of genes found with the core bacteria to the total bacterial community, the values were tightly correlated (*r*=0.984). However, 21 gene families showed statistically significant differences (*t*-tests, Bonferroni-corrected *P*<0.05) as indicated in Figure 3. We compared the predicted functions of the complete communities from salamanders exposed to a soil reservoir with salamanders without a reservoir. We illustrate significant differences in biosynthesis of secondary metabolites, which tended to be higher in the salamanders in the soil reservoir microbiota, and immune system gene functions (level 3 KOs), which tended to be higher in the salamanders without a soil reservoir (Supplementary Figures 2 and 3).

## Discussion

Host microbiota performs a number of important functions for their hosts, such as disease resistance (Dillon *et al.*, 2005; Rosenberg *et al.*, 2007; Harris *et al.*, 2009a, 2009b; Becker and Harris, 2010), metabolism, vitamin production, development and activity of the immune system and behavior (Turnbaugh *et al.*, 2007). In this study, we examined the bacterial community dynamics on healthy, red-backed salamanders that were not infected with *Bd*. It is likely that the stability and diversity of host microbiota are related to the consistency and quality of protection. In both the soil and the sterile media treatments, the community composition of the salamander skin microbiota changed when brought into the laboratory (Figure 1a). Thereafter, alpha diversity remained stable over the course of the experiment for salamanders maintained in the presence of a soil bacterial reservoir. These results suggest that relatively minor disturbances, such as skin sloughing (Meyer *et al.*, 2012), which occurred during the experiment in the laboratory, do not affect diversity if a bacterial reservoir is available. In addition, we identified a stable core set of eight OTUs that were consistently present across all treatments and time points.

In the field, there was no correlation between alpha diversity of salamanders' skin microbiota and the microbiota of the salamanders' immediate habitat upon capture in the field.

Thus, even though their microbes are environmentally derived, salamander skin communities are not determined solely by passive inoculation. Rather, host factors appear to select for and maintain host-associated microbes at similar relative abundance over time. The same pattern has been found in humpback whales and has been suggested to occur in amphibian larvae (Apprill *et al.,* 2011; McKenzie *et al.,* 2011). The strong exception here is the relative abundance of the uncultured Opitutuae in the sterile media treatment. Further work characterizing this bacterium will be necessary to determine whether it is specifically associated with amphibians or whether the increase in relative abundance (up to 90% of the community) seen in the sterile media condition is a result of the Opitutuae opportunistically taking advantage of microbe-poor laboratory conditions. If so, it would suggest that its abundance is not controlled by host factors under laboratory conditions.

In the laboratory, alpha diversity decreased in the sterile media treatment; however, remained constant in the soil treatment. This result suggests that a diverse bacterial reservoir, such as soil, supports the presence of a large number of relatively rare or transient bacterial species that may compete with the core microbes and suppress their abundance. When salamanders are housed in soil, and in the wild, they are in constant contact with transient bacteria that are attempting to colonize the salamanders. We propose that some bacteria have either a mutualism or commensalism with amphibians and are adapted to live on their skin, and their abundance and persistence are likely influenced by host factors, such as the secretion of antimicrobial peptides. These symbionts are interacting with the transient bacteria, and some transients will be good competitors. Competition with immigrant bacteria and steady disturbance from skin shedding would support high diversity (as seen in the soil treatment). On the contrary, when the salamanders do not have a bacterial reservoir containing transients (sterile media), immigration of new OTUs ceases. This could lead to competitive release as competition between symbionts and transients would not occur, resulting in lower diversity and the potential of one species to become relatively dominant, as we saw with the uncultured Opitutuae.

An alternative, but not mutually exclusive, explanation is that some bacteria need to be regularly seeded on an amphibian to be common, and therefore they become less common without a reservoir. It is unlikely that the initial stress from capture and placement into captivity caused a decrease in diversity because of the new conditions favoring a few bacterial species, as we saw consistent diversity in the presence of a soil reservoir and a slow decline of diversity in the sterile media treatment rather than a sharp initial drop (Figure 2). However, captive conditions without soil may be more stressful than housing with soil. In the future, a sterile soil treatment can test whether changes in diversity were because of the presence or absence of a diverse bacterial reservoir rather than the presence or absence of soil *per se*. However, bacterial communities of the salamanders were more similar to the bacterial communities of their respective substrate (Figure 1b), suggesting that the bacterial diversity in the soil was a determinant of bacterial diversity on salamanders rather than the presence or absence of soil itself. Therefore, it appears that in the natural environment, the soil is an important source of bacteria but that host factors sculpt the structure and diversity of these communities. The maintenance of diversity is important. Indeed, diversity suppressed disease susceptibility in a tropical frog species (Bell 2013), as found in other systems (Dillon *et al.*, 2005; Verhulst *et al.*, 2011). In addition, *P. cinereus* had greater morbidity if their bacterial diversity was experimentally reduced using antibiotics and hydrogen peroxide prior to *Bd* exposure (Becker and Harris, 2010).

The relative abundance of the core microbiota and alpha diversity was negatively correlated. When communities became less diverse through time, the relative abundance of the core increased and in some cases composed as much as 93.5% of the community. A novel, dominant core OTU from the phylum Verrucomicrobia and class Opitutuae comprised as much as 92.5% of the bacterial community in the sterile media (Table 2). Verrucomicrobia is commonly found within intestines (van Passel *et al.*, 2011b) and soil (Bergmann *et al.*, 2011; van Passel *et al.*, 2011a). This phylum has been under-represented because of PCR bias (Bergmann *et al.*, 2011), which may have affected detection in earlier studies of amphibian systems. Interestingly, Verrucomicrobia (*Akkermansia*) in the human gut degrades mucin and became dominant after a disturbance because of antibiotic therapy (Dubourg *et al.*, 2013). In the present study, the role of Opiutae is unknown; however, this OTU became dominant in this study after the major disturbance of captivity and lack of a soil bacterial reservoir. The increase in the relative abundance of the core in the absence of a bacterial reservoir may be because the core species are among the most abundant bacteria before the perturbation, and therefore are more likely to increase.

Many of the core OTUs are known to have antimicrobial activity. Five of the eight core OTUs were in the family Pseudomonadacae. The most common genus in this family is *Pseudomonas,* and it is commonly found on amphibians' skin and have been shown to be antifungal (Lauer *et al.,* 2007). This genus is a known probiotic in other systems such as agriculture (Pierson and Weller, 1994; Hass and Défago, 2005). Another of the core OTUs is in the family Staphylococcaceae; the most studied genus within the family is *Staphylococcus* and is commonly cultured from human skin (Dworkin, 2006). Another betaproteobacterium, a member of the family Comamonadaceae, was in the core community, and this family was previously found to be abundant on amphibians (McKenzie *et al.*, 2011). The last core OTU is a novel Opitutuae, which has been discussed above. These results indicate that bacterial groups that are readily cultured from amphibians are part of the core and are common members of the community.

The core bacterial community may be important in maintaining essential functions. When comparing the predicted functions of the core community to the entire community, we found that the functions are highly correlated. This suggests that the core community is responsible for functions that are important for the symbiosis to persist. However, it is important to note that the function of the currently unculturable Verrucomicrobia is unknown and therefore not in this analysis. In addition, there were differences in predicted gene functions between the core and non-core communities, which suggest that core and non-core communities have unique functional roles. The PICRUSt analysis generated two hypotheses: (1) keeping amphibians with an environmental reservoir of bacteria increases or maintains bacterial diversity and the diversity of secondary metabolites associated with the skin ecosystem (Figure 3, Supplementary Figure 3). The core bacteria may produce abundant antifungal metabolites if they are co-evolved with the host. Experiments are needed to test the effect of diversity on host health; (2) the core bacteria may be tightly linked with immune regulation (Supplementary Figure 3), and functions involved in immune evasion may be most prevalent in core bacteria and these bacteria could be activating or deactivating these host-defense pathways (Zocco *et al.*, 2007; Thekkiniath *et al.*, 2013).

Our results show that captivity and animal husbandry conditions affect skin microbiota, which may have implications for amphibian microbial ecology experiments that are conducted in the laboratory and for captive-breeding programs (Becker *et al.*, 2009; Harris *et al.*, 2009a, 2009b; Becker and Harris, 2010). We have found that the microbiota changed upon entering captivity and that the diversity of the bacterial communities is likely dependent on the availability of a bacterial reservoir in soil; however, this may be different for aquatic amphibians that are not in as constant contact with soil. In many of the experiments on the role of bacteria protecting amphibians against *Bd*, the amphibians have been housed in containers with sterile artificial pond water (Becker *et al.*, 2009; Harris *et al.*, 2009a, 2009b; Becker and Harris, 2010; Becker *et al.*, 2011), which are conditions similar to our sterile media treatment. Therefore, these amphibians may have been more susceptible to disease than they would have been in nature. In addition, our findings that the skin microbiota changes under traditional captive conditions may be relevant to captive-rearing programs where animals are raised in pristine conditions with the intentions of being released into the wild. For instance, captive-rearing programs are occurring in the United States of America for hellbenders (*Cryptobranchus alleganiensis*), boreal toads (*Anaxyrus boreas boreas*), mountain yellow-legged frog (*Rana muscosa*) and chiricahua leopard frogs (*Lithobates chiricahuensis*) (Muths *et al.*, 2001; Fellers *et al.*, 2008; Soorae, 2011; Bodinof *et al.*, 2012, respectively). These amphibians are also likely to have depauperate and atypical microbiota, as they have no natural bacterial reservoir. It is important to determine how captivity affects the microbiota of animals in repatriation programs in order to establish natural or protective microbiota prior to release. Restoration from atypical and depauperate bacterial communities may be possible using the protective communities found on wild, healthy animal, and further research is needed. Indeed, probiotic therapy is a promising disease-mitigation strategy (Bletz *et al.*, 2013), which should be considered as a part of animal husbandry practices.

## Figures and Tables

**Table 1:** Salamander sampling scheme in the laboratory

*Day of experiment*	*Numbers of salamanders sampled*	
	*Housed with soil*^a^	*Housed with sterile media*^a^
0	10 (10)	10 (9)
7	10 (4)	10 (5)
14	10 (4)	10 (6)
21	10 (7)	10 (7)
28	10 (8)	10 (6)

a Numbers in parentheses represent the number of samples that amplified and are included in the analysis.

**Table 2:**
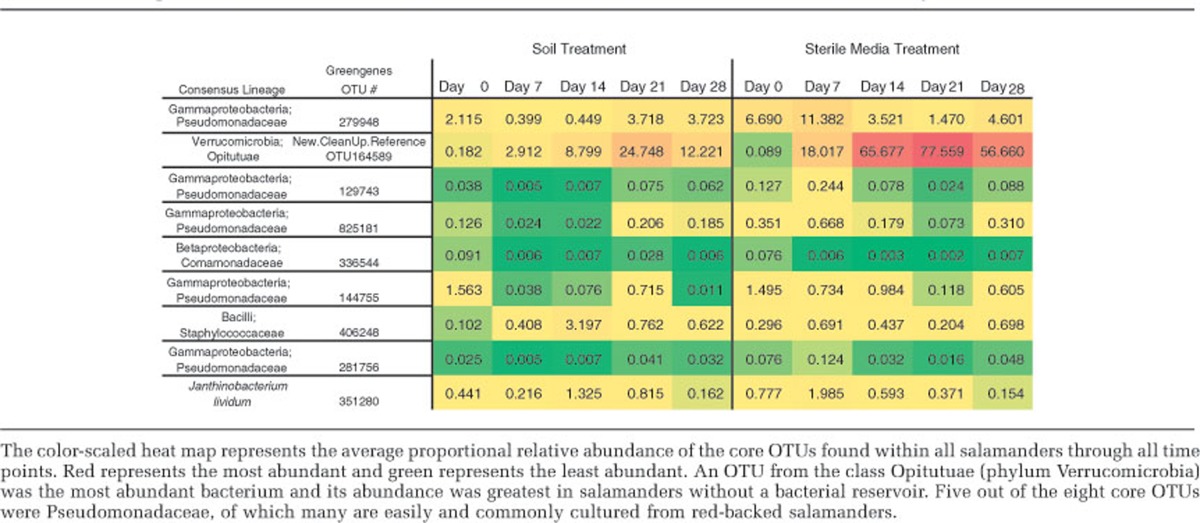
Heat map of the core OTUs and the *Janthinobacterium lividum* OTU for each treatment through time
