# Supplementary material for: Microbial community dynamics and effect of environmental microbial reservoirs on red-backed salamanders (Plethodon cinereus)
Source: ISME J. 2013 Dec 12;8(4):830–40. doi: 10.1038/ismej.2013.200 (PMC3960541; doi:10.1038/ismej.2013.200)
Supplement: Supplementary Information [file ismej2013200x1.doc]

**Supplementary Information**

**Contents:**

**Supplementary Figures 1–3.**


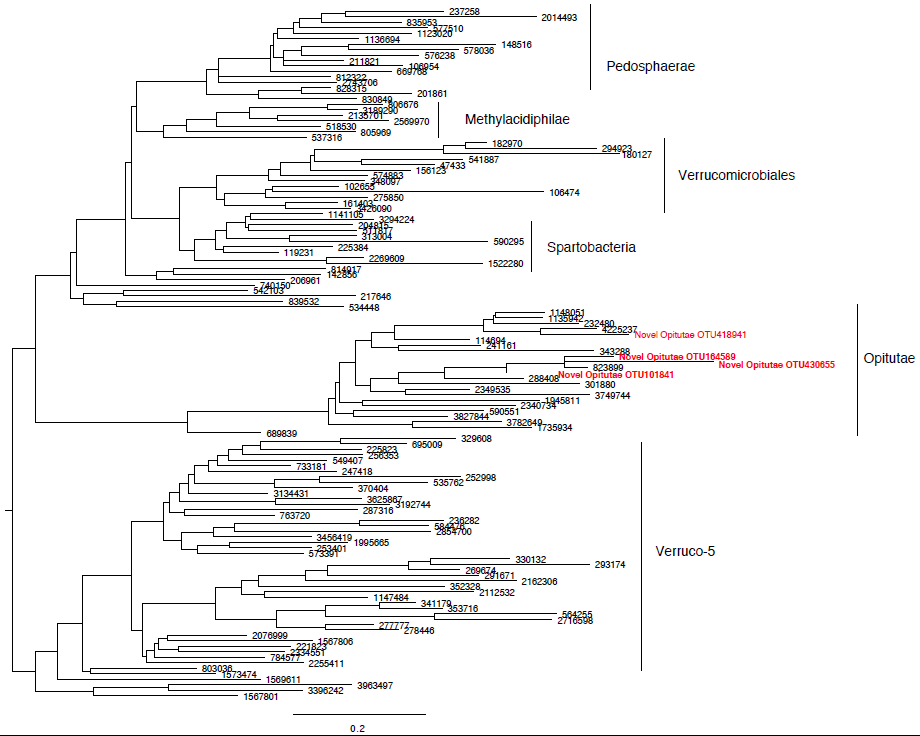


**Supplementary Figure 1.** Phylogenetic tree constructed by placing novel Verrucomicrobia sequences within the Greengenes tree (filtered to sequences with 85% similarity; obtained from http://greengenes.secondgenome.com/downloads/database/12_10 using the EPA algorithm within RAxML {Berger, 2011}, including the OTU that corresponds to the highly abundant member of the redback salamander core community (OTU 418941). These sequences are robustly placed within the Verrucomicrobia class Opitutae. The tree is labeled with Greengenes prokMSA ids.

**Supplementary Figure 2.** Relative abundance of genes involved in biosynthesis of secondary metabolites predicted from salamander skin bacterial communities on day 28 of the experiment. * indicates gene categories that are significantly different (t-test, Bonferroni-corrected *P* < 0.05), and higher on salamanders in the soil reservoir treatment compared to the sterile media treatment.

**Supplementary Figure 3.** Relative abundance of genes involved in immune system processes predicted from salamander skin bacterial communities on day 28 of the experiment. * indicates gene categories that are significantly different (t-test, Bonferroni-corrected *P* < 0.05), and higher on salamanders in the sterile media treatment compared to the soil reservoir treatment.
